# Evaluation of Effects of Diabetes Mellitus, Hypercholesterolemia and Hypertension on Bell’s Palsy

**DOI:** 10.3390/jcm10112357

**Published:** 2021-05-27

**Authors:** George Psillas, Grigorios G. Dimas, Anastasia Sarafidou, Triantafyllos Didangelos, Vasilios Perifanis, Georgia Kaiafa, Daphne Mirkopoulou, Thomas Tegos, Christos Savopoulos, Jiannis Constantinidis

**Affiliations:** 1 1st Academic ENT Department, Aristotle University of Thessaloniki, AHEPA Hospital, 1, Stilponos Kyriakidi St., 546 36 Thessaloniki, Greece; tesisaraf@gmail.com (A.S.); janconst@otenet.gr (J.C.); 2 1st Propaedeutic Department of Internal Medicine, Aristotle University of Thessaloniki, AHEPA Hospital, 1, Stilponos Kyriakidi St., 546 36 Thessaloniki, Greece; gregorydimas@yahoo.gr (G.G.D.); didang@auth.gr (T.D.); bperifanis@yahoo.gr (V.P.); gdkaiafa@auth.gr (G.K.); daphnemircopoulou@gmail.com (D.M.); chrisavopoulos@gmail.com (C.S.); 3 1st Department of Neurology, Aristotle University of Thessaloniki, AHEPA Hospital, 1, Stilponos Kyriakidi St., 546 36 Thessaloniki, Greece; ttegos@auth.gr

**Keywords:** facial nerve, facial palsy, prognosis, risk factors, recovery rate

## Abstract

The aim of this study is to evaluate the effects of diabetes mellitus, hypertension and hypercholesterolemia on the clinical presentation and outcome of Bell’s palsy. The study (comorbidity) group consisted of 50 patients with Bell’s palsy associated with diabetes, hypertension, or hypercholesterolemia; the control group included 46 patients with Bell’s palsy, but without comorbid diseases. The House–Brackmann grading system (I to VI) was used in order to assess the initial and final facial functions. Both groups of patients were treated with steroids and the antiviral agent acyclovir. The mean severity of initial facial paralysis was more significant in diabetes, hypercholesterolemia, and hypertension, in comparison to the control group. Patients suffering from Bell’s palsy and concomitant comorbidities have a poorer prognosis (HB III-VI) compared to patients without comorbidities. Increased glycosylated hemoglobin A1c levels (>6.7%) were significantly correlated with unsatisfactory facial recovery. The pathogenetic mechanisms by which diabetes, hypercholesterolemia, and hypertension affect the vasa nervosum of facial nerve have been described.

## 1. Introduction

Bell’s palsy (BP) is the most common form of acute facial nerve paralysis, with an annual incidence of about 20–30 per 100,000, and a lifetime incidence of one in 60–70 people [1,2].

The etiology of BP implicates infective, immune, and ischemic mechanisms; among them, the viral hypothesis remains the most plausible, implying reactivation of neurotropic herpes viruses 1 (HSV-1), 2 (HSV-2), and Varicella zoster virus (HZV) at the level of geniculate ganglion [3,4]. Association with other infectious pathogens, such as cytomegalovirus, Epstein–Barr virus, mumps, rubella, and human immunodeficiency virus has also been shown [5]. In addition, common immune pathways have been advocated for BP and Guillain–Barré syndrome, a cell-mediated, autoimmune neuritis [5]. Moreover, microcirculatory disorders induced by atherosclerosis have been reported to increase the accumulation of lipids in the intima of vasa nervosum of facial nerve, resulting in vascular inflammation, luminal obstruction, and ischemia [1]. As the endoneurial pressure increases, damage by either direct mechanical injury or by reducing blood flow occurs from compression of the narrowest portion of the bony facial canal [6].

Many clinical parameters have been evaluated as prognostic factors in patients with BP [7,8,9]; in this study, we focused on the effect of diabetes mellitus (DM), hypertension (HT), and hypercholesterolemia (HC) on the clinical presentation and outcome of BP.

## 2. Materials—Methods

**Figure 1:**
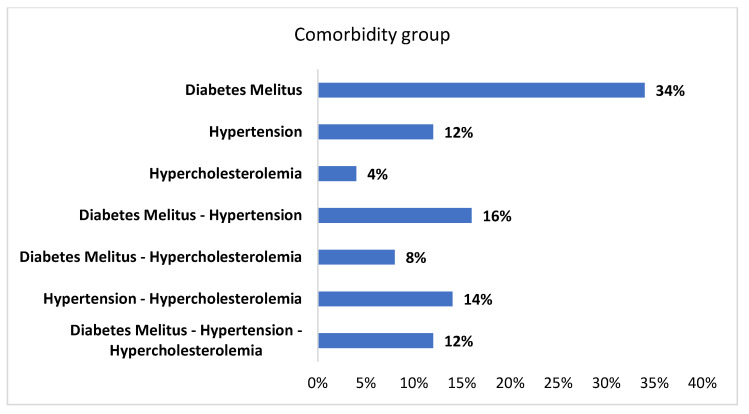
Distribution of diabetes mellitus, hypertension, and hypercholesterolemia in the comorbidity group suffering from Bell’s palsy.

**Figure 2:**
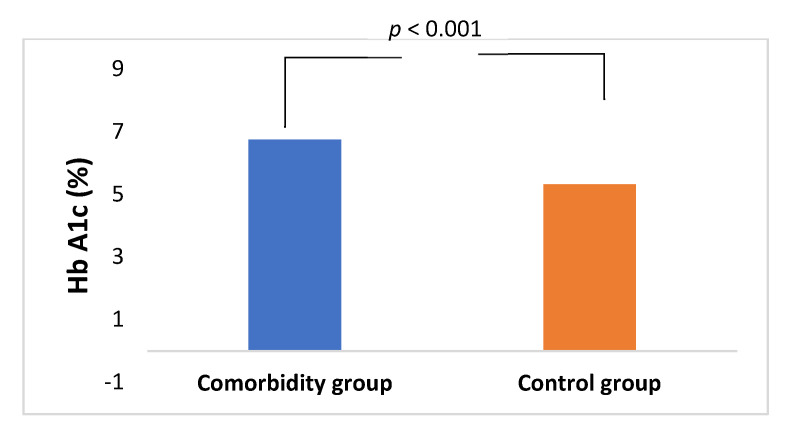
Glycosylated hemoglobin Hb A1c (%) in the comorbidity and control group suffering from Bell’s palsy.

This retrospective study enrolled 96 patients who presented with Bell’s palsy (BP) to the emergency ENT department over the last 4 years. Among these patients, 50 (20 males, 30 females, aged 46–89 years old, mean 65.1 ± 13.4) (Table 1) were identified as having diabetes mellitus (DM) and/or hypertension (HT), and/or hypercholesterolemia (HC) and were selected for the comorbidity group (Figure 1). The diagnosis criteria were defined according to the American Association’s criteria. These criteria are, more specifically, DM (fasting plasma glucose ≥ 126 mg/dL, 7.0 mmol/L) as demonstrated by glycosylated hemoglobin HbA1c > 6.0% [10], HT by blood pressure ≥ 130/80 mmHg [11], and HC by total cholesterol > 200 mg/dL, LDL > 130 mg/dL and HDL < 40 mg/dL [12]. Those patients who declared being diabetic, hypertensive, and having hypercholesterolemia before BP onset were included in our study. One patient (60 y, female) had DM type I. The other 46 patients (28 males, 18 females, aged 39–87 years old, mean 57.6 ± 12.4) with BP, but without comorbidities diseases, were used as the control group (Table 1). Mean values of HbA1c for the comorbidity and control group are shown in Figure 2.

The House–Brackmann (HB) grading system (Table 2) was used to assess the facial function at admission and last examination during the follow-up until complete recovery (range: 3–9 weeks, median: 5 weeks), or 6 months after the onset of BP for those with incomplete recovery (HB III–VI). The HB grade I was defined as complete recovery, HB II as mild facial palsy, HB III–IV as moderate facial palsy, and HB V–VI as severe facial paralysis.

Both groups were treated within 48 h from BP onset with intravenous dexamethasone (Decadron 8 mg × 3/day with schema for one week), intravenous acyclovir (Zovirax 250 mg × 2/day) for 3 days, and then per os (500 mg × 4/day). For DM patients, antidiabetic drugs and/or insulin with dose adjustments were given, and the blood pressure of HT patients was regularly measured. Before treatment, all patients in the first group had reported adequate therapeutic control of their conditions.

## 3. Statistic Methods

Statistical analysis was conducted with the use of SPSS v.25.0 software (SPSS, Chicago, IL, USA). The distribution of variables was evaluated using the Shapiro–Wilk test. Continuous variables are expressed as means ± standard deviation, and categorical variables as percentage frequencies. Comparisons of continuous variables were conducted with either *t*-test for independent samples or the Mann–Whitney *U* test. The homogeneity of variances was tested using Levene’s test. Categorical variables were compared using the x^2^ and Fisher Exact test. A *p*-value of <0.05 (Student’s *t*-test) was set as the limit of the statistical significance of the tests. 

## 4. Results

In our study, 34 (35%) out of 96 patients with BP were identified as suffering from DM, 27 (28%) patients from HT, and 19 (19%) patients from HC. Before treatment of BP, the mean severity (HB grade) of initial facial paralysis was more significant in DM (*p* < 0.001), HC (*p* < 0.01), HT (*p* < 0.05), and in DM combined with HT (*p* < 0.05), HC (*p* < 0.01), and HT-HC (*p* < 0.01), compared to the control group (Table 3). Moreover, at admission, patients with DM, HT, and HC presented with moderate facial palsy at lower proportions (28%) compared to the control group (41.2%), but with severe facial palsy at higher rates (62% vs. 32.5%) (Table 4). Hypertension, HC, and association of all comorbidities (DM, HT, and HC) in one patient were more common in females affected by BP (*p* < 0.05) (Table 3); the mean age was found to be higher in DM (*p* < 0.01), HT (*p* < 0.05), in DM associated with HT (*p* < 0.05), and HT-HC (*p* < 0.05) (Table 3).

At posttreatment follow-up, our results showed that when DM, HT, and HC, or DM-HT, DM-HC, HT-HC, and DM-HT-HC were compared to controls, no significant effect (*p* > 0.05) was found on the final HB grade, from mild to severe facial palsy (Table 3). However, an unsatisfactory outcome (HB grade III to VI) of BP was more pronounced in the comorbidity group (28%) than in the control group (8.7%) (Table 4). Complete recovery was achieved in 20 (58.8%) out of 34 patients with DM, 19 (70.3%) patients with HT, 14 (73.6%) with HC, and 8 (57.1%) with DM associated with HT (Table 3).

In Table 5, the recovery distribution of facial function to the different HB grades for the two groups is illustrated; e.g., in the comorbidity group, from the 16 patients with initial HB V, 8 patients recovered to final HB I, 2 patients to HB II, 5 to HB III and 1 patient remained in HB V. It was noted that 13/31 (42%) patients of the comorbidity group with initial severe BP (HB V and VI) did not have a final satisfactory recovery (HB ≥III) (Table 5); for the control group, 26% (4/15) of patients with initial severe BP did not have satisfactory recovery, which was quite lower. Moreover, the multivariable analysis has shown that the increased glycosylated hemoglobin A1c levels (>6.7%) was significantly correlated with unsatisfactory facial recovery (HB ≥ III) (Table 6).

In total, 68 (70.8%) out of 96 patients with BP had complete recovery to HB I (Table 4). A slight female predominance (51.5%) was found in terms of complete recovery to normal facial function, and their ages ranged from 40 to 87 (mean 66.56 ± 11.66 years). However, males and females had equal recovery to HB ≥2 (aged 39–89, mean 60.57 ± 12.10). Therefore, our statistical analysis did not reveal any statistical significance for age (*p* = 0.896) and gender (*p* = 0.455) in relation to the final HB grade (HB I/HB II-VI).

Among patients with incomplete recovery, 2 out of 14 patients in the comorbidity group and 2 out of 4 in the control group developed synkinesis at least 5 months after the onset of BP.

## 5. Discussion

A high proportion of DM (35%), HT (28%), and HC (19%) was detected in our patients with BP, which was greater than their incidence in the general population. More specifically, in recent studies, the percentage of DM in the general population has been estimated to be 8.5% [13], approximately 8.5% for HT [14], and 12% for HC [15]. However, the frequency of patients with DM affected by BP in the literature varies extensively, i.e., 11–39% [2,3,4,7,16,17,18,19,20], 18–46% for HT [2,3,4,7,16,19], and 6.7–28% for HC [16,20].

It has been supported [18,21] that the severity of facial palsy at the onset of BP does not differ significantly between patients with DM and without DM. On the other hand, Riga et al. [16] reported that severe BP (HB grade V, VI) was significantly more frequent (70%) in patients with DM, but they did not find any statistical correlation between HT and HC with the severity of BP. According to our results, it is possible that DM (*p* < 0.001), but also HT, HC, and the association of all comorbidities (DM, HT, and HC) in one patient may affect the initial severity of BP (Table 3). Karyia et al. [6] have demonstrated that the vessel walls of the bony facial nerve canal (vasa nervosum) were found to be thicker in DM (type 1 and 2) patients compared with normal controls, suggesting that DM patients are more vulnerable to ischemia and severe facial palsy; this also contributes to the inflammation and edema of the facial nerve in BP, which leads to the entrapment and strangulation of the facial nerve within the bony canal [6].

At the onset of BP, the mean age of our patients (Table 1 and Table 3) was higher than that of our control group, and the mean age of other studies, i.e., 60.8 years [22] and 56.1 years [23]. Similarly, in Șevik Eliçora and Erdem [21], the mean age differed significantly between the diabetic group (62.8 years) and the nondiabetic group (47.5 years), with both groups being affected by BP. It has also been reported that DM and HT may increase the risk of BP among older patients [3,19]. However, the fact that the mean age of patients suffering from BP was older in the comorbidity group than the control group could be the result of the increased likelihood to suffer from DM or HT at older rather than younger ages.

The possible effect of DM, HT, and HC on the facial nerve outcome in BP remains controversial. In more recent literature, some authors have demonstrated that there was no correlation between DM [2,7,9,16,21,24], HT [1,2,9,16,24], HC [2,16], and final recovery. By contrast, DM was shown to be a poor prognostic factor for favorable recovery after BP [1,4,8,18,20]. Instead of DM, there is less evidence in literature for HT being a cause of BP [3,24]. Our results showed that when DM, HT, HC, or DM-HT, DM-HC, HT-HC, DM-HT-HC were compared to controls, no significant effect was found on the final HB grade from mild to severe palsy (Table 3). However, the overall rate of incomplete recovery for BP (HB III-VI) was statistically larger for the comorbidity group compared to control group (Table 4). Thus, patients presenting with BP and associated comorbidities have to be followed more closely and would profit from an intensified therapy (a higher steroid dose with larger insulin dose adjustments), although high steroid treatment may disturb the A1c levels. The poor prognosis in the comorbidity group may be due to the coexistence of vasculopathic factors (e.g., DM, HT, HC), and the associated microangiopathy, which would lead to microcirculatory failure of the vasa nervosum, resulting in facial nerve ischemia and possibly infarction [17]. This pathogenetic mechanism can explain our finding that five (10%) patients of the comorbidity group had severe (HB IV-VI) persistence of BP, and none in the control group (Table 5).

Few studies have reported on the rate of facial nerve recovery after BP in relation with underlined comorbid diseases, such as DM, HT, and HC. Kanazawa et al. [18] found that 52.6% of 19 diabetic patients had complete recovery (HB I), which was lower than 58.8% of our 34 diabetic patients. However, Riga et al. [16] showed higher rates of recovery, i.e., 85% (vs. 58.8%) of 20 patients with DM, 84.6% (vs. 70.3%) of 26 patients with HT, and 81.2% (vs. 73.6%) of their 16 patients with HC. Jung et al. [1], in their group of patients recovered to HB I and suffering from BP and concomitant comorbidities (DM, HT and HC), have demonstrated somewhat higher rates of recovery than our findings. Described as complete recovery were HB I combined with HB II, i.e., 75.9% (vs. 70.5% in our study by adding HB I plus II) for patients with DM, 90.7% (vs. 74%) for HT and 88.1% (vs. 84.2%) for HC. By contrast, Kim et al. [2] reported complete recovery at lower proportion compared with our group (47.3% vs. 64%) for BP and adjacent comorbidities. In any case, our results showed low rates of facial nerve recovery after BP in patients with comorbidities, and this might be attributed to the negative effect of vasculopathic factors, such as DM. The glycosylated hemoglobin A1c levels were found to be increased (>6.7%) and were significantly correlated with unsatisfactory facial recovery (HB ≥ III) (Table 6). It has been reported that hyperglycemia can cause direct facial nerve injury by several mechanisms including increased oxidative stress, accumulation of advanced glycation endoproducts, and impaired flow through the polyol pathway [23]. Similarly, electrophysiological tests, such as the blink reflex have shown considerable delays on both sides of the face in the late stages of BP in DM, supporting the assumption that DM has negative effects on the outcome of peripheral facial palsy [22]. Peitersen [25] in his study describing the spontaneous course of 2500 cases of peripheral facial palsy, reported that the recovery for patients with DM was very poor, and only 25% achieved normal facial nerve function; he supported that the poorer prognosis resulted from a vascular insufficiency and diabetic polyneuropathy.

As DM is often associated with polyneuropathy, it was questioned whether BP is part of a subclinical systemic polyneuropathy. Benatar and Edlow [26] reported that BP patients may harbor additional cranial neuropathies, such as trigeminal, glossopharyngeal, or hypoglossal neuropathy. Moreover, Chaco [27] studied 30 patients with Bell’s palsy and found low conduction velocity in median and ulnar nerves in 14 of these patients; however, larger studies will be required to determine this association.

The association between severe HT and BP has been essentially described in children and pregnant women [3,28]; pathogenic mechanisms included swelling of the facial nerve in its bony canal, hemorrhages into the facial canal or facial nerve nucleus, and ischemic strokes affecting the postnuclear fibers of the nerve [28]. Bell’s palsy usually occurred during exacerbation of severe arterial HT due to nonadherence of medication and was immediately resolved after successful blood pressure control, suggesting that controlled HT was a factor of favorable facial function outcomes [4,28].

Synkinesis, which are abnormal, involuntary associated facial movements, have been reported as sequelae of BP and usually occur from 12 to 54 weeks (mean 24.8 weeks) [29]. Synkinesis increases with the degree of palsy, and BP starts to recover 3 months after the onset [25,29]. In our study, 4 (4.1%) out of 96 patients with BP developed synkinesis at least 5 months after the onset; however, this incidence may have been underestimated, since our follow-up stopped after 6 months and did not continue any longer than this.

## 6. Conclusions

Among comorbid diseases, diabetes mellitus may affect the initial presentation and outcome of Bell’s palsy more commonly than hypertension and hypercholesterolemia. Patients suffering from BP and concomitant comorbidities may have poorer prognosis (HB III-VI) compared to patients without comorbidities, necessitating closer follow-up and intensified therapy.

## Figures and Tables

**Table 1:** Mean age by gender of patients suffering from Bell’s palsy for the comorbidity and control group.

Mean Age (Years) ± SD	Comorbidity Group	Control Group	Statistic Test
Male	65.9 ± 9.7	59.1 ± 13.3	t(46) = 1.933, *p* = 0.059
Female	64.6 ± 9.9	55.4 ± 11.1	t(46) = 2.983, *p* < 0.01
Total	65.1 ± 9.7	57.7 ± 12.5	t(94) = 3.272, *p* < 0.01

**Table 2:** House–Brackmann classification of facial nerve function.

Grade	Description	
I	Normal	Normal symmetrical function.
II	Mild dysfunction	Slight weakness noticeable only on close inspection. Complete eye closure with minimal effort. Slight asymmetry of smile with maximal effort. Synkinesis barely noticeable, contracture or spasm is absent.
III	Moderate dysfunction	Obvious weakness, but not disfiguring. May not be able to lift eyebrow. Complete eye closure and asymmetric mouth movement with maximal effort; obvious but not disfiguring synkinesis.
IV	Moderate severe dysfunction	Obvious disfiguring weakness; inability to lift brow; incomplete eye closure and asymmetry of mouth with maximal effort; severe synkinesis.
V	Severe dysfunction	Motion barely perceptible; incomplete eye closure, slight movement of corner of mouth; synkinesis.
VI	Total paralysis	No movement; loss of tone; no synkinesis, contracture or spasm.

**Table 3:** Correlation of diabetes mellitus (DM), hypertension (HT), hypercholesterolemia (HC), and DM and HT with gender, age and initial—final House–Brackmann (HB) grade in patients suffering from Bell’s palsy. * Statistical significance (*p* < 0.05).

	DM (*n* = 34)	HT (*n* = 27)	HC (*n* = 19)	DM & HT (*n* = 14)	DM & HC (*n* = 4)	HT & HC (*n* = 7)	DM & HT & HC (*n* = 6)	Control Group (*n* = 46)
Gender (male/female)	16/18 (Χ^2^(1) = 1.065, *p* = 0.302)	8/19 (Χ^2^(1) = 5.759 *p* < 0.05) *	5/14 (X^2^(1) = 5.640 *p* < 0.05) *	6/8 (X^2^(1) = 1.088, *p* = 0.297)	2/2 (X^2^(1) = 0.181, *p* = 0.670)	1/6 (Χ^2^(1) = 5.321, *p* < 0.05) *	0/6 (Χ^2^(1) = 7.913, *p* < 0.05) *	27/19
Age (Mean ± SD)	66.82 ± 9.72 (t(78) = 3.137, *p* < 0.01)	65.56 ± 8.08 (t(71) = 2.540, *p* < 0.05) *	64.84 ± 10.35 (t(63) = 1.887, *p* = 0.064)	68.29 ± 7.37 (t(58) = 2.686, *p* < 0.05) *	65.25 ± 15.327 (t(48) = 1.147, *p* = 0.257)	63.14 ± 7.669 (t(51) = 1.122, *p* = 0.267)	68.50 ± 8.503 (t(50) = 2.054, *p* < 0.05) *	58.57 ± 12.87
Initial HB grade (Mean ± SD)	4.82 ± 1.29 (t(78) = 4.097, *p* < 0.001)	4.26 ± 1.51 (t(71) = 2.090, *p* < 0.05) *	4.74 ± 1.41 (t(63) = 3.040, *p* < 0.01)	4.71 ± 1.44 (t(58) = 2.641, *p* < 0.05) *	5.75 ± 0.500 (t(48) = 2.876, *p* < 0.01)	3.86 ± 1.773 (t(51) = 0.589, *p* = 0.559)	5.33 ± 0.516 (t(50) = 2.871, *p* < 0.01)	3.48 ± 1.56
Final HΒ grade (HB I/HB II-VI)	20/14 (Χ^2^(1) = 3.517, *p* = 0.061)	19/8 (Χ^2^(1) = 0.570, *p* = 0.450)	14/5 (Χ^2^(1) = 0.159, *p* = 0.690)	8/6 (Χ^2^(1) = 2.448, *p* = 0.118)	2/2 (Χ^2^(1) = 1.611, *p* = 0.204)	7/0 (Χ^2^(1) = 1.876, *p* = 0.171)	4/2 (Χ^2^(1) = 0.402, *p* = 0.526)	36/10

**Table 4:** Initial and final House–Brackmann (HB) of Bell’s palsy in the study group (comorbid diseases: diabetes mellitus, hypertension, and hypercholesterolemia), and the control group.

HB Grade	Comorbidity Group (*n* = 50)	Control Group (*n* = 46)	
I-II	36 (72%)	42 (91.3%)	X^2^(1) = 5.861, *p* < 0.05
III-VI	14 (28%)	4 (8.7%)	

**Table 5:** Recovery distribution of facial function in Bell’s palsy to the different House–Brackmann (HB) grades (A) in the study group (comorbid diseases: diabetes mellitus, hypertension, and hypercholesterolemia) and (B) in the control group.

A	Final HB (Comorbidity Group, *n* = 50)						B	Final HB (Control Group, *n* = 46)					
Initial HB	I	II	III	IV	V	VI	Initial HB	I	II	III	IV	V	VI
II	5						II	11	1				
III	11						III	15	1				
IV	2		1				IV	2	1				
V	8	2	5		1		V	6	2	1			
VI	6	2	3	3		1	VI	2	1	3			

**Table 6:** Correlation of final House–Brackmann (HB) grades and glycosylated hemoglobin HbA1c, hypertension, and hypercholesterolemia with multiple logistic regression analysis—odds ratio (95% confidence interval). * Statistical significance (*p* < 0.05).

Final House–Brackmann Grade	I–II	III–VI	
HbA1c > 6.7%	9	7	*p* = 0.004 *, OR = 3.5 (1.2–13.5)
Hypertension	22	7	*p* = 0.2, OR = 1.2 (0.2–5.1)
Hypercholesterolemia	16	3	*p* = 0.3, OR = 0.3 (0.4–7.1)

## Data Availability

The data presented in this study is available upon request from the corresponding author.
